# Blood in Dropsies with Albuminous Urine

**Published:** 1846-06

**Authors:** 


### ARTICLE XVII.

### BLOOD IN DROPSIES WITH ALBUMINOUS URINE.

In the Abstract in our last number upon the Physiology and Pathology of the Blood, want of space compelled us to defer, for the time, the consideration of that important pathological condition of this fluid, in which there is a diminished amount of the albumen of the serum.

*Dropsical effusions*, as has been proven by numerous investigations upon this subject, are, generally, coincident with this abnormal condition of the blood.

This being admitted as a fact, we are naturally led to inquire why it is that a tendency to dropsy follows thus, a loss of albumen?

We may obtain some light upon this point by inquiring into the composition of the effused fluids, and into the general condition of the system in dropsies. Investigations upon this subject show, "that the serosity which has been effused, even while remaining composed of the same materials as the serum of the blood, contains, proportionally, more water than this fluid, and much less of the organic principles, particularly albumen."

In thirty-six analyses of dropsical fluids made by Andral, the maximum amount of albumen was 48, and the minimum 4,—68 or 70 being the normal proportion in the blood.

The relative amount of albumen in these fluids, seems not to be influenced either by the seat or cause of the disease; the variation seemed rather to depend upon the general condition of the patient. In proportion to the varying strength of the constitution, albumen increases or diminishes. In cases of hydrocele, where the constitution remains comparatively strong and healthy, the amount of albumen varies from 35 to 59, while, on the other hand, in ascites and other dropsies, attended with much general debility, the amount of this constituent is small.

It may be remarked, also, that the amount of albumen in the fluids, effused from inflamed surfaces, is large in proportion to the activity of the inflammatory action. The above facts seem to indicate that impoverished and thin blood passes more readily through relaxed and debilitated tissues, and that these combined pathological conditions of the solids and fluids are the immediate causes of dropsical effusions.

As to the remote causes of these abnormal conditions, Andral remarks that "cases of dropsy following insufficient alimentation have been cited, and Dr. Graspard has even reported a true epidemic of this kind, that prevailed in 1816, through several departments of the interior of France, as a result of a great scarcity which had afflicted those districts. The inhabitants had been reduced to seek their food among the roots and herbs of the fields. A large number of them became dropsical. History informs us that the same thing has occurred at other epochs, under the influence of the same circumstances. It is probable that in these singular epidemics, the insufficiency of alimentation must have modified the composition of the blood; that there was the point of departure of the dropsy, and it is allowable to conjecture that the blood under the empire of this influence experiences a diminution of its albumen."

Dropsical effusions and deficiency of albumen are generally simultaneous pathological conditions. In some cases, however, the cause of dropsy seems to act mechanically, by obstructing the circulation and thus causing congestion and a consequent effusion, of the blood's more fluid constituents, through the coats of its vessels. Examples of the kind are presented in cirrhosis of the liver, preventing the passage of blood from the portal veins, thus causing ascites; and in such cardiac diseases, as of the valves &c., which prevent the free return of blood from the systemic veins, producing general dropsy.

But of all the diseases concomitant with dropsical effusions, those of the kidneys, producing albuminous urine and a consequent diminution of the blood's albumen, are the most common and important. We will now, therefore, give a brief synopsis of the more recent and generally adopted views of pathologists upon this class of diseases.

We will premise, however, that in order fully to comprehend the changes, caused by disease in the structure or function of an organ, it is important that we should first thoroughly understand its healthy organization and physiological action. We have given, therefore, in the preceding article a brief history of the minute anatomy of the kidney, and of the more modern views with regard to its functions.

Dropsical effusions with albuminous urine, may be said, in general terms, to depend upon a diseased condition of the kidney, by which the free circulation of blood through its capillaries is prevented. Upon referring to the structure of these glands, it is evident that the blood in its course from the renal arteries to the emulgent veins, must pass through both the Malpighian and portal plexuses, the former being within the cyst-like dilitation which communicates directly with the tubuli uriniferi, the latter external to and surrounding the walls of these excretory tubes. It is evident, then, that an obstruction to the passage of blood through the portal plexus would cause an accumulation and congestion to take place in the Malpighian tuft.

The texture of the vessels forming the Malpighian plexus, being thin and delicate, and peculiarly favouring the passage of fluids; in the diseases under consideration, and in consequence of such a congestion, the serum of the blood exudes from them into the uriniferous tubes, and in cases of long continued and much congestion, the pressure is often such as to produce a rupture of the coats of these vessels, and a consequent escape of other constituents of the blood.

Albuminous urine then, is indicative of an amount of congestion in the Malpighian plexus, sufficient to cause an exudation of serum, of which albumen is one of the principal ingredients, through the coats of these capillary vessels. And whenever red globules appear in the urine, it is an evidence of rupture of the walls of these delicate tubes.

The serum of the blood may escape and produce albuminous urine, either with or without this laceration of the vessels. When, however, this fluid has a smoky appearance, indicating the presence of blood capuscles, or shows indications of fibrine, by the presence of coagulated fibrinous moulds of the tubuli, we have unmistakeable evidence of rupture of the capillaries of the Malpighian plexus.

It appears, then, that these abnormal constituents of the urine in these cases, are not secretions from the kidney, but proximate elements of the blood, which have escaped from vessels, ruptured by congestion, produced by obstruction to the circulation in the portal plexus.

This obstruction, according to Dr. Johnson, is produced by the accumulation of epithelium cells in the tubuli, distending and pressing them together so as to prevent the free passage of blood through the portal capillaries by which they are surrounded. This accumulation may take place suddenly and rapidly, producing congestion and the consequence, acute or inflammatory dropsy; or gradually and slowly, and in connection with fatty depositions, as in fatty kidney or Bright's diseases.

The first mentioned and more acute form of renal disease is generally caused by irritating matters retained in the blood, as for instance the cutaneous excretions, not eliminated when the functions of the skin have been checked by disease or exposure to cold. For as the cutaneous and renal functions are similar and at times reciprocal, the irritiatng matters not excreted by the skin, in such cases, seek a passage from the system through the kidneys, causing increased action, irritation, congestion, and a morbid generation and accumulation of epithelium cells.

The following case, reported by Dr. Todd, will serve to illustrate.

"A woman 25 years of age was admitted to the Hospital in the sixth month of her pregnancy. She had always enjoyed good health till about a month ago, when she caught cold, and became troubled with cough and hoarseness. A fortnight after this, she observed a swelling of the labia pudendi and of the legs; her urine became scanty, and in it she noticed a red sediment. When she came into the hospital there was considerable œdema of the labia, and of the lower extremities, accompanied with some fluctuation of the abdomen. The urine was scanty, smoky, and highly acid; it was turbid, and abundantly albuminous; it contained lithate of ammonia, and had a sp. gravity of 1.027. The microscope was not used; but the presence of blood was sufficiently evinced by the smoky and albuminous character of the urine. The skin was dry. In this case I endeavored to promote the healthy action of the skin by the use of warm baths, and by the administration of Dover's powder and antimony. Under this treatment the albumen soon disappeared from the urine, and the dropsy from the legs, and in twelve days all the symptoms that had previously been present had vanished."

"In these classes of cases, the aim of the treatment must be to restore the healthy action of the skin, to relieve congestion, to purify the blood of noxious ingredients by promoting the various excretions. How far the treatment to be pursued may be antiphlogistic, or more or less stimulant and tonic, must depend in a great degree on the peculiar circumstances of each particular case."

It has already been stated that there are certain affections, during the course of which, or subsequently, the dropsical diseases under consideration often appear. During the continuance, or after an attack of scarlet fever, for instance, dropsical effusions with albuminous urine frequently supervene, producing fatal effects often, when the original disease has been of the mildest form. Facts leading to more correct pathological views with regard to these cases, are, therefore, interesting and of great practical importance.

Why is it, then, that dropsy with albuminous urine, follows so frequently an attack of scarlet fever?

It is a well known fact, that the "scarletina poison" effects, by its morbific influence, all the organs of the body, producing general irritation, congestion, and a depressed condition of the vital powers. Among other effects, it is evident that the healthy functions of the skin must be greatly impaired, if not entirely suspended, thus causing an increased flow of morbific matter to the kidneys, and adding to the irritating and depressing influence already acting upon these organs. It is also true that exfoliation takes place, not only of the cuticle from the integuments covering the body, but also of epithelium scales, from the mucous membranes, lining cavities, and excretory tubes.

It seems, then, that coincident with, and following an attack of scarlet fever—the morbific cause of this disease, irritating matters not excreted by their proper organs, increased action, and a tendency to the production of an exuberance of epithelium, are all causes acting upon the kidneys to produce congestion and rupture of their Malpighian capillaries, and as the consequence, dropsical effusions and albuminous urine.

The following is a case in point:

"A boy, five years old, has had scarlet fever, which seems to have run its usual course. About three weeks after the abatement of the fever he became universally dropsical. His urine was found to be scanty in quantity, smoky in colour, presenting a dark appearance, as if mixed with carbonaceous matter. On the application of heat, or of nitric acid, it yielded an abundant precipitate of albumen. Under the microscope other constituents of the blood were plainly seen, namely, blood discs and fibrinous casts of the uriniferous tubes. There was also a large quantity of lithic acid. The skin was dry, not freely secreting, and some fever was present. In order to promote the action of the skin, I employed the warm-bath and sudorifics, liq. ammon. acet. with nitre, and as the presence of blood in the urine indicated congestion of the kidney, a few leeches were applied to the loins. Not three days have passed, and the dropsy is almost cured. The urine is more copious, less albuminous, and less smoky."

The treatment in cases like the above, should be upon the same general plan as that previously recommended.

There is still another most formidable disease, which, though slower in its progress, and differing in its pathology from those already described, produces, in the blood and urine, the same pathological changes.

From what has been said of the diseases already described, it appears that in cases following exposure to cold and other causes, which act promptly and suddenly, the obstruction to the circulation, in the portal plexus, is caused by an accumulation of epithelium cells in the tubes of the kidney, in unusual quantities, but normal in quality. In Bright's disease, on the other hand, the obstruction is caused, not by an accumulation of healthy epithelium cells, but by a morbid deposition of fat distending and filling their cavities.

It appears, from recent investigations upon this subject, that a small quantity of fatty matter is naturally deposited in the epithelium cells of the kidney, and that its abnormal accumulation, distending these cells and filling the tubuli, obstructs the circulation in the capillaries of the portal plexus, and thus causes congestion of the Malpighian tuft, albuminous urine, dropsical effusions, and at length suppressed secretion, followed by all the fatal effects consequent upon retention of urea in the circulating fluid.

With regard to the accession and progress of Bright's disease, it may be remarked that it presents in its course three distinct stages, indicating a greater or less deposition of fatty matter.

The symptoms, in the first stage, are remarkable for their abscurity, and for the absence of any marked indications of renal disease. The most prominent symptoms are those of dyspepsia, imperfect assimulation secretion and excretion, with indications of deficiency of the blood's globular element.

These signs of disease are, often, so slight, however, as not to attract the attention of the patient or his friends, and even, when a physician is called, he finds no certain indication of the disease in this insipient stage, unless by a microscopical examination, fatty epithelium cells are discovered in the urine.

The second stage, being that in which medical advice is generally first sought, is characterized by pain in the loins, pallid countenance, edema of face and lower extremeties, dyspepsia, frequent micturition, especially at night, and urine albuminous or showing the presence of other ingredients of the blood, and in all cases containing fatty epithelial cells.

The disease, in this stage, is, often, of such an acute character as to lead to its being attributed to inflammation.

In the third and last stage the dropsical effusions are general, there is anasarca and often ascites; the urine is albuminous, and its secretion imperfect and in small quantity, consequently, there is a retention of urea in the blood, giving rise to epilepsy, fatal coma, and other cerebro spinal affections, indicative of its poisonous effects.

The pathological changes indicative of Bright's disease, are irregular vascular congestions, of the structure of the Kidney, the vessels being full at some points, and empty at others, giving a tuburculated and mottled appearance to the surface of the organ. The true and never failing pathological change in Bright's disease, however, is the presence of epithelium cells filled with fat, and distending the tubuli.

This fatty deposition in the cells of the kidney is produced evidently by similar, if not the same causes, which give rise to fatty liver, in which there is an accumulation of the same matter, in the epithelial cells of this organ.

"The frequent association of fatty liver with fatty kidney strongly indicates that the two diseases are closely allied to each other, and owe their existence to a similar, or even to the same cause. There is no evidence that this disease is inflammatory. Fat is certainly not a product of inflammation, nor do we find any of the inflammatory products in the kidneys in this disease. No pus, no gangrene, no lymph, in the substance of the kidney. To explain the disease, we must look to those circumstances which favour the formation of fat; deficiency of oxygen, want of exercise, impure air, and the like.

"Mr. Simon and Dr. Johnson have artificially produced the disease. They kept cats for some time in the dark, causing them to breathe impure air, and to live on unwholesome food. The results were, that they were enabled to trace the disease through its three stages. They drew off the urine from the bladder by a catheter, and examined it from time to time; they found that first fat began to appear in the urine, with numerous gorged cells. There was soon a tendency to dropsy, and the urine became smoky and albuminous. The dropsy increased, the urine became scanty, and death from suppression followed.

"In the treatment of the disease, we must look to the particular stage in which we find it. If perchance the patient applies for advice in the very earliest stage, and the physician is fortunate enough to detect it, the disease may be cured. If, before mechanical obstruction and its consequences have arisen, the mode of life and the habit of the person be changed by pure air and careful attention to diet and exercise, then it is quite possible that the coming evil may be averted. Unfortunately, however, a patient seldom applies for medical aid thus early.

"In the second and third stages we can only palliate suffering, by relieving congestion, promoting the flow of urine, and the action of the skin; and perhaps we may check the tendency to the abnormal deposit of fatty matters, by restricting him to a diet which contains neither fat nor butter, nor any of those nonazotized substances which are nearly allied in chemical constitution to fat, and are easily convertable into it—such as starch, sugar, potatoes, &c. We must not allow fat, butter, and such things, to be taken, nor any of those substances which, like potatoe, starch, &c., are easily convertable into fat."

